# Evolution of Advanced Chronic Lymphoid Leukemia Unveiled by Single-Cell Transcriptomics: A Case Report

**DOI:** 10.3389/fonc.2020.584607

**Published:** 2020-10-30

**Authors:** Pavel Ostasov, Henry Robertson, Paolo Piazza, Avik Datta, Jane Apperley, Lucie Houdova, Daniel Lysak, Monika Holubova, Katerina Tesarova, Valentina S. Caputo, Iros Barozzi

**Affiliations:** ^1^ Laboratory of Tumor Biology and Immunotherapy, Biomedical Center, Faculty of Medicine in Pilsen, Charles University, Pilsen, Czechia; ^2^ Department of Surgery and Cancer, Imperial College London, London, United Kingdom; ^3^ Imperial BRC Genomics Facility, Imperial College London, London, United Kingdom; ^4^ Centre for Haematology, Department of Immunology and Inflammation, Imperial College London, London, United Kingdom; ^5^ NTIS, Faculty of Applied Science, University of West Bohemia, Pilsen, Czechia; ^6^ Department of Haematology and Oncology, University Hospital Pilsen, Pilsen, Czechia; ^7^ Faculty of Medicine in Pilsen, Institute of Medical Genetics, Charles University in Prague and Faculty Hospital, Pilsen, Czechia; ^8^ Hugh & Josseline Langmuir Centre for Myeloma Research, Centre for Haematology, Department of Immunology and Inflammation, Imperial College London, London, United Kingdom

**Keywords:** ****chronic lymphoid leukemia (CLL), single-cell RNA-seq (scRNA-seq), therapy resistance, disease progression, advanced disease, case report

## Abstract

Genetic and transcriptional heterogeneity of Chronic lymphocytic leukaemia (CLL) limits prevention of disease progression. Longitudinal single-cell transcriptomics represents the state-of-the-art method to profile the disease heterogeneity at diagnosis and to inform about disease evolution. Here, we apply single-cell RNA-seq to a CLL case, sampled at diagnosis and relapse, that was treated with FCR (Fludarabine, Cyclophosphamide, Rituximab) and underwent a dramatic decrease in CD19 expression during disease progression. Computational analyses revealed a major switch in clones’ dominance during treatment. The clone that expanded at relapse showed 17p and 3p chromosomal deletions, and up-regulation of pathways related to motility, cytokine signaling and antigen presentation. Single-cell RNA-seq uniquely revealed that this clone was already present at low frequency at diagnosis, and it displays feature of plasma cell differentiation, consistent with a more aggressive phenotype. This study shows the benefit of single-cell profiling of CLL heterogeneity at diagnosis, to identify clones that might otherwise not be recognized and to determine the best treatment options.

## Introduction

Chronic lymphocytic leukaemia (CLL) is the most common form of adult leukaemia in Western countries and accounts for approximately 30%–40% of all leukemias (1). It is a disorder of B cells characterized by the accumulation of small, mature-appearing lymphocytes in the blood, the bone marrow, and lymphoid tissues.

The clinical presentation of CLL is very heterogeneous. The progression ranges from an aggressive course requiring treatment early after diagnosis to cases with indolent behavior that do not need therapeutic intervention for many years. While CLL can be classified into two subgroups based on mutational status of immunoglobulin heavy-chain variable region gene (IGHV) (2), abnormal changes also include several genetic mutations and chromosomal aberrations, expression of microRNAs (3) and epigenetic changes (4). Chromosomal abnormalities such as del(13q), del(11q), del(17p), or trisomy 12 are present in 80% of the cases (5). Somatic mutations have been detected in genes with roles in DNA damage (*TP53* and *ATM*), mRNA processing (*SF3B1*), Notch signaling (*NOTCH1*) and inflammatory pathways (*MYD88*) (6).

Available treatments include chemotherapy (either alone or combined with immunotherapy), targeted therapies such as venetoclax (targeting BCL-2) or ibrutinib (targeting Bruton’s tyrosine kinase), and CAR-T cells (7). Currently, the standard therapy combines Fludarabine with anti-CD20 antibody (Rituximab) and Cyclophosphamide (FCR).

CLL cells typically coexpress the surface antigen CD5 together with the B-cell antigens CD19, CD20, and CD23. The immunophenotype of CLL lymphocytes correspond to an intermediate stage of B-cell maturation. CD19 is exclusively expressed throughout B-cell development from the proB-cell stage until the plasma cell stage, which is CD19 negative.

Prognostic factors derived from high-throughput genetic and phenotypic profiling can potentially identify patients who require therapy relatively soon after diagnosis and are at higher risk for disease progression. Whole exome sequencing of matched pre-treatment and relapse samples detected the presence of a resistant clone at pre-treatment in 30% of the cases (8). On the other hand, analysis of an ibrutinib-treated cohort of patients failed to identify a significantly higher risk of relapse in individuals with baseline mutations affecting either the BTK pathway or TP53 (9). These studies suggest that sub-clonal genetic alterations at diagnosis can be selected by the therapy but are not sufficient to explain the relapse, pointing to an important role for non-genetic factors. This advocates for profiling transcriptional heterogeneity at diagnosis using single-cell approaches, which can shed light on possible evolutionary trajectory of each individual tumor. We here report a case of CLL which underwent FCR and presented with a relapse enriched for CD19-negative cells. The loss of CD19 was completely spontaneous (without any selective pressure induced from pharmacological treatments with anti-CD19 drugs) and it resulted in a more aggressive behavior. By applying single-cell transcriptomics, we were able to identify the presence of this CD19-negative clone at diagnosis, albeit at low frequency. This clone shows unique biological features that would be hindered by bulk-RNA-seq, highlighting the strength of single-cell-RNA-seq in predicting the possible progression trajectories and therefore inform therapeutic strategies.

## Case Report

### Case Description

A 58-years-old female without previous oncological or other serious illness was diagnosed with MALT lymphoma of the parotic gland in 2013 (extranodal lymphoma of the marginal zone; WHO classification 2008). PET-CT revealed involvement of equilateral cervical and supraclavicular lymph nodes (Ann Arbor stage IIE). The examination of the bone marrow also showed the presence of a CLL clone. CLL presented as early stage disease (Rai stage 0-I, nodular and interstitial infiltration of the bone marrow, ALC 9.0*10^9^/L). Cytogenetic and IgVH mutation status evaluations were not performed. Considering the dominant clinical presentation of MALT lymphoma and that the criteria for initiation of treatment against CLL were not met, the patient was treated according to the protocol for lymphomas, consisting of six cycles of R-CHOP (cyclophosphamide, doxorubicin, vincristine, prednisone, and rituximab). Complete remission of lymphoma was achieved (CT, Cheson criteria 2014), with the residual CLL population still detectable in the bone marrow (10^-2^, assessed by flow cytometry).

**Figure 1:**
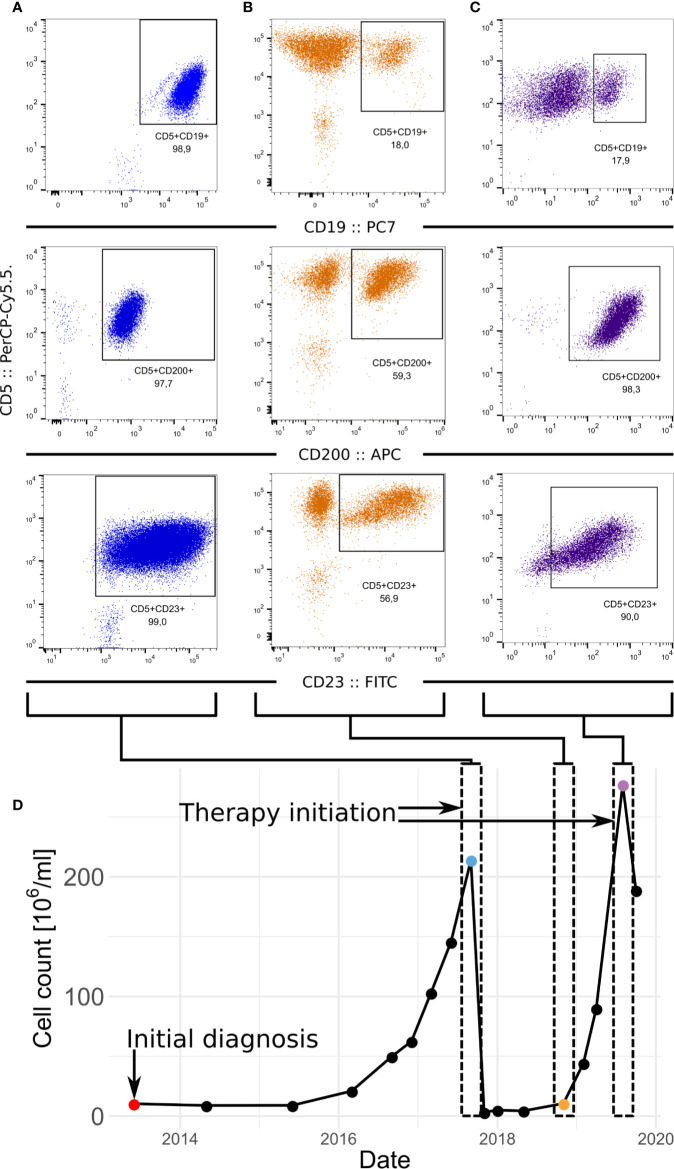
Development of the CLL in patient. **(A–C)** FACS of samples at diagnosis **(A)** relapse **(B)** and before the second therapeutic intervention **(C)**. CLL fraction of cells in the sample is defined as fraction of CD5-positive CD23-positive cells which is supported by fraction of CD5-positive CD200-positive cells. **(D)** Progression of the numbers of leukocytes in the peripheral blood in time.

Slow haematological progression (estimated by blood count) of CLL was observed from 2015 to 2017. At that time, CLL progressed to advanced disease (Rai III) and met the criteria for initiation of therapy (lymphocyte doubling time of 5 months, anaemia, disease-related symptoms). FISH highlighted deletions of chromosome 14 (98% of evaluated cells), and of 13q, 14q, and 17p (harbouring TP53) in about 5% of interphase nuclei. At this time, the first sample (termed diagnosis) was collected and examined by flow cytometry (see **Supplementary Methods**). This showed that virtually all CLL cells (CD200-, CD5-, and CD23-positive) were CD19-positive (**Figure 1A**).

The patient received six cycles of chemoimmunotherapy (FCR; rituximab: 375mg/m^2^; cyclophosphamide 250mg/m^2^; fludarabine 25mg/m^2^) which resulted in partial remission (bone marrow not evaluated). At the end of 2018, the patient relapsed (clinical stage Rai III). At this time point the second sample was collected (peripheral blood, termed relapse) and a CD19-negative sub-clone was detected by flow cytometry (**Figure 1B**). The proportion of all CLL cells to CD19-positive cells was 3:1. The cytogenetic analysis detected an expansion of the complex karyotype clone including 17p deletion (FISH; >90% of nuclei). The disease showed an increased ratio of all CLL cells to CD19-positive cells to 5:1, suggesting further progression of the CD19-negative clone (**Figure 1C**). A timeline highlighting disease progression and sampling is shown in Fig 1D. At this point, the patient met the iwCLL criteria for initiating treatment (anaemia, disease-related symptoms). Considering the TP53 status, targeted therapy with ibrutinib was initiated. An allogeneic stem cell transplantation was also considered for future consolidation.

After 3 months of this monotherapy (420 mg/day), the patient was investigated for neurological symptoms (expressive aphasia). A meningioma was discovered on MRI scan (left frontal lobe, total size 29 mm x 30 mm x 28 mm). Ibrutinib treatment was stopped, and the tumor (meningothelial meningioma, grade I, WHO 2016) was surgically removed. Unfortunately, 2 months later the patient died of complications (cryptococcal meningitis, hydrocephalus and ischemic stroke). At that time, CLL response to the treatment corresponded to stable disease with significant peripheral lymphocytosis (80x10^9^/L, ANC > 5x10^9^/L, and IgG level 4,4 g/L).

### Single-Cell Transcriptomics Highlights a Shift in Clones’ Dominance in Advanced CLL

**Figure 2:**
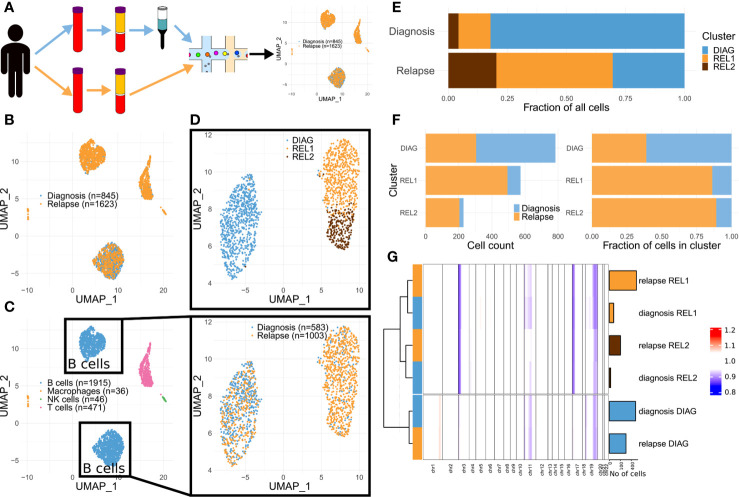
Single-cell transcriptomics highlights a shift in clones’ dominance in advanced CLL. **(A)** Schematics of the approach. **(B)** Dimensionality reduction showing the captured single-cell transcriptomes (diagnosis and relapse). **(C)** Same as **(B)** but color-coded according to predicted cell types. **(D)** Dimensionality reduction applied to the cells in the B cell compartment from **(C)** (upper panel: color-coded by cluster, see Methods; lower panel: color-coded by diagnosis vs relapse). **(E)** Stacked bar charts indicating the relative cluster composition of the cells sampled either at diagnosis or relapse. **(F)** Stacked bar charts indicating both the absolute (left) and relative (right) composition of each cluster, in terms of cells sampled either at diagnosis or relapse. **(G)** Summary heat map showing the average CNA profile for each indicated subgroup (n = 6; for each one of the three clusters in **(D)**, two groups were defined, one comprising the cells at diagnosis and another one those at relapse). Each column of the heat map represents a proxy for the number of copies for the region, estimated using a genomic windowing approach (chr1 to chr22), based solely on the scRNA-seq data. Bar charts on the right side of the heat map indicate the size of each group. Dendrogram indicate the results of hierarchical clustering performed using complete linkage and Ward.D distance.

After de-multiplexing the diagnosis and relapse samples based on their specific barcode (**Figure 2A**), we obtained 1,062 and 2,026 cells, respectively (**Table S1**). For further analyses we retained 845 and 1,623 cells from diagnosis and relapse passing filtering criteria, respectively. We then subject them to dimensionality reduction (**Figure 2B**) and graph-based clustering using Seurat (10) (see **Supplementary Methods**). This resulted into 7 clusters (**Figure S1A**), which were then assigned to known cell types using signatures from CIBERSORT (11) (see **Supplementary Methods**). Three clusters, encompassing most of the cells, were classified as B cells, two clusters as T cells, and the two smallest clusters as NK cells and macrophages, respectively (**Figures 2C**, **S1A**, and **S1B**). We then re-run the dimensionality reduction and clustering steps considering only cells from clusters classified as B cells. This resulted in three clusters (**Figure 2D**). While one of these clusters was dominated by cells from diagnosis (termed DIAG or diagnosis-enriched cluster), two of them were mainly composed by cells from relapse (REL1 and REL2; hereafter termed relapse-enriched clusters) (**Figures 2E, F**).

In order to track the clonal evolution at the genetic level, we then estimated large chromosomal alterations directly from the scRNA-seq data. The copy number alterations (CNAs) profile of each single cell was estimated using InferCNV (12) (see **Supplementary Methods**). Visual inspection of the profiles revealed consistent differences between cells in the diagnosis- vs. relapse-enriched clusters (**Figure S1C**). This prompted us to generate meta-profiles for each of the clusters, separately for the cells from the diagnosis and relapse samples. Hierarchical clustering showed that the meta-profiles aggregate based on their malignancy status (the clusters defined in **Figure 2G**) rather than their origin (diagnosis or relapse). Those cells at diagnosis that are showing both similar CNA and transcriptome to majority of cells at relapse suggest the pre-existence of at least one, less represented clone at diagnosis, that underwent expansion upon treatment. This clone shows specific loss of chromosomes 3p and 17p (**Figures 2G** and **S1C**), confirming previous cytogenetic analysis at later collection. Loss of one copy of *TP53* (which sits on 17p) was previously associated to progression to advanced, drug resistant CLL (5, 13, 14). The loss of 17p was also linked to altered pharmacokinetics of rituximab (15).

### Activation of Specific Pathways in the Relapse-Enriched Clone

**Figure 3:**
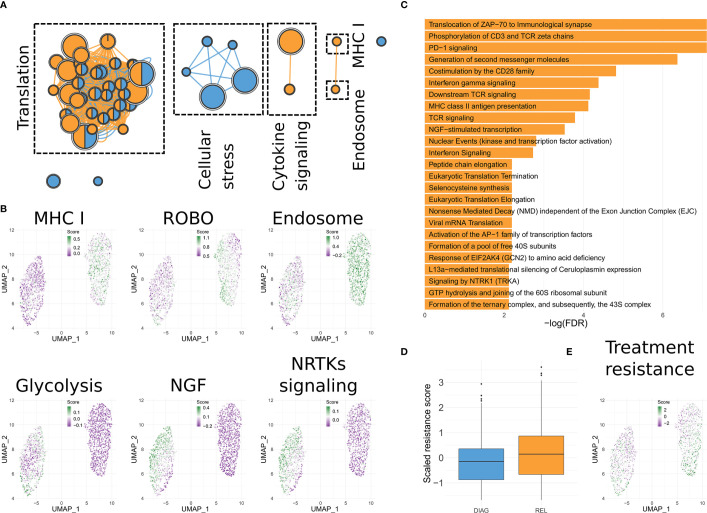
Functionality of the differentially expressed genes. **(A)** Enrichment Map representation of the Reactome pathways significantly enriched in genes up-regulated in the relapse-enriched clone. Each node is a pathway. Size of the node is proportional with the number of genes associated to it. Nodes are connected based on similarity and represented as pies, indicating if DEGs annotated in the pathway that are either up- or down- regulated in the relapse-enriched clone. **(B)** UMAP visualization of B cells as shown in **Figure 1D**, in which each cell has been color-coded according to the cumulative expression of the genes in the indicated gene set. **(C)** Bar charts showing the statistically significant enriched Reactome pathways in the lists of DEGs between the sub-clusters of the relapse-enriched cluster. **(D)** Box plots indicating the overall expression of a published signature predictive of time-to-progression in CLL for combined Rituximab, Fludarabine and Cyclophosphamide treatment (17), for each of the two main clusters identified. **(E)** Same as **(C)** but using the signature from **(D)**.

We then asked if genes related to specific functions could explain the resilience of this clone to several cytotoxic and targeted treatments. To this aim, we derived the differentially expressed genes (DEGs; FDR <= 0.05) between the relapse-enriched and the diagnosis-enriched clusters (90 up-regulated and 85 down-regulated; **Table S2**). Enrichment analysis highlighted dozens of Reactome pathways significantly associated with either the up- or down-regulated genes in the relapse (FDR <= 0.05; **Table S3**). To achieve a compact representation of the enriched terms, we used Enrichment Map (16) (**Figure 3A**). Both up- and down-regulated genes in relapse show over-representation of terms related to different aspects of translation. Several enriched pathways are linked to an increased motility in the relapse-enriched clusters (e.g. ROBO signaling; **Figure 3B** and **Table S4**). Those up-regulated show also enrichment for antigen presentation (MHC class I) and cytokine signaling (**Figures 3A, B** and **Tables S3**, **S4**). The latter has been previously associated to advanced disease (18). In contrast, down-regulated genes showed enrichment for signaling pathways related to cellular stress (**Figure 3A**). Of note, these analyses also suggested a metabolic difference between the diagnosis-enriched and relapse-enriched clones, as hinted by enrichment in glycolytic pathways in the former (**Figure 3B**).

### Further Investigation of the Heterogeneity of the Relapse-Enriched Clone

Clustering of B cells data resulted in two main groups, with the one enriched for cells from the relapse sample divided into two sub clusters (**Figure 2D**). We then determined the DEGs between REL1 and REL2 (n = 55; **Table S5**). Reactome enrichment analysis revealed that these DEGs are significantly enriched in signaling pathways that are related to different aspects of B cells functionality like CD28, ZAP70, or PD-1 signaling (**Figure 3C**), and in pathways already identified when comparing the diagnosis-enriched to the entire relapse-enriched cluster (**Figure 3B**, **Tables S3** and **S4**). This suggests further heterogeneity in the expression of these pathways at relapse. Interestingly, one of the genes significantly up-regulated in REL2 vs. REL1 is *CXCR4*. This gene has been shown to confer leukemic cells a stronger ability to survive, migrate, and interact with the microenvironment (19). In line with the predicted *TP53* loss, testing for enrichment of TP53 target genes (20, 21) among either up- or down-regulated genes in the relapse-enriched clones, revealed a statistically significant overlap only with the down-regulated genes (FDR <= 0.05, hypergeometric test).

### A Signature of Bone Marrow Plasma Cells Characterizes the Relapse-Enriched Clone

Given these differences between the diagnosis-enriched and relapse-enriched clones, and this further heterogeneity within the latter, we asked if these mirrored characteristics of the physiological differentiation hierarchy of B cells. In other words, we tested if we could ascribe part of the observed variation to transcriptional features characterizing different stages of B cell maturation. Using data from GenomicScape (22), we assigned each B cell to known stages of differentiation (see **Supplementary Methods**), then summarized the results by cluster (**Figure S1D**). This analysis showed a significant over-representation (*p*-value < 2.2e-16 for REL1 vs. DIAG and *p*-value = 2.4e-12 for REL2 vs. DIAG; Fisher’s exact test) of cells with bone marrow plasma cells (BMPCs) signature in the relapse-enriched clone. This suggests that cells in this clone might have a more quiescent phenotype than the dominant clone before treatment, which is in line with the functional enrichment of the DEGs (**Figures 3A-C**).

### The Expression Profile of the Relapse-Enriched Clone Predicts Survival and Resistance to Treatment

We then systematically surveyed PRECOG (23) to highlight if any of the genes found as DEGs between the relapse-enriched and the diagnosis-enriched clones was highly predictive of patient survival. Indeed, we identified four genes (*PARP14, STAT1, UCP2, IFT57*) showing higher expression in the relapse-enriched clone that were significantly associated to poor prognosis in patients showing high expression (FDR <= 0.05). Interestingly, the gene that showed the highest significance was *PARP14* (**Figure S1E**). This has been previously reported to play a role in chemoresistance (17). Our analysis also highlighted one gene (*ZNF292*) showing lower expression in the relapse-enriched clone and that was significantly correlated to poorer prognosis of tumors displaying low expression. *ZNF292* has also been previously reported as inactivated in 2%–3% of CLL cases, and it has been included in the list of CLL driver genes (24).

Next, we took advantage of a published signature predictive of time-to-progression in CLL for the same treatment that this patient underwent (FCR) (25). Those cells in the relapse-enriched clone (particularly those in REL2) showed indeed higher levels of expression for the genes in this signature, compared to the cells in the diagnosis-enriched clone (*p*-value = 2.1e-12; Wilcoxon rank-sum test; **Figures 3D, E**).

## Discussion

Despite the availability of several treatment options (26), the heterogeneity of CLL still limits the ability to eradicate the residual disease and prevent relapse.

In this case report we show how a relatively low-throughput single-cell transcriptomics profile at diagnosis can be highly informative. This approach highlighted a major switch in clones’ dominance during treatment, with one of the clones prevailing at diagnosis and one at relapse. As opposed to scRNA-seq, standard flow cytometry could not distinguish these two clones at the time of diagnosis. Data analysis revealed the distinctive activated pathways and propensity to chemoresistance of these two clones (**Figure 3**) and that the clone that persisted upon therapy presented features of differentiated plasma cells and would rather match plasma cells disorders.

CLL is a plastic disease that can develop resistance to treatments by many phenotypic changes like the downregulation of CD20 or CD19 (27). CD19 negativity is often observed in relapse after immunotherapy using CAR-T (7). It has been speculated that loss of CD19 could be connected to a lineage switch caused by mutation of *CD19* or its alternative splicing, which precludes its detection (28, 29). In the presented case, loss of CD19 was not directly connected to lineage switch but we observed differences that could be linked to a progression between stages of B cells differentiation. The fraction of CD5-positive/CD19-positive cells was initially almost 100% but minor clones (clusters REL1 and REL2 in our analysis) were already represented (**Figure 2E**), although showing detectable CD19 on their surface. At relapse, the CD5-positive/CD19-positive cells were reduced to 18%, which is in line with the results from scRNA-seq (**Figure 2E**). This suggests that the initially dominant clone retained CD19 expression while the relapse-enriched clusters lost it. Such a drop in CD19-positive cells is rarely observed in CLL (30). In B cell differentiation, CD19 is present at all stages except for plasma cells. The cells in the relapse-enriched clone showed a significant over-representation of the BMPCs signature, thus indicating a plasma cell like phenotype. The most common plasma cell disorder—multiple myeloma—is CD19-negative in almost all cases (31). While cases of concomitant or sequential CLL and multiple myeloma are rare we could not confirm the presence of multiple myeloma in this patient. Nonetheless, non-neoplastic CD19-negative, long-lived memory plasma cells have been previously identified in healthy donors (32). Although all CLL cells (the diagnosed and the relapsed) were CD20-positive (immunophenotyping; not shown) and plasma cells are usually CD20-negative, about 20% of plasma cells in healthy individuals as well as in myeloma patients can express it (33). Besides, we identified a mild but statistically significant reduction of the CD20 gene (*MS4A1*) at the mRNA level in the relapse-enriched clone (**Table S2**). The differentiation into plasmablasts is also accompanied by a switch to an oxidative metabolism (34), which we observed in the relapse-enriched clone.

Comparison of the genetic and transcriptional profiles of single cells in the two clones suggests pre-existent differences correlated with resistance to treatment (**Figure 2**). Among these, and in line with the loss of chromosome 17p (which harbours TP53) in the relapse-enriched clones, we detected down-regulation of TP53 targets in the relapse clones. Loss of *TP53* leads to increased resistance to apoptosis which could be further enhanced by other factors like interleukin-12 (IL-12) (35). Interestingly, we observed upregulation of genes related to IL-12 activity in the relapse-enriched clone. Moreover, IL-12 expression is correlated with advanced Rai stages (18). Another factor that might have further protected cells from apoptosis is the higher expression of *PARP14* (36).

## Concluding Remarks

The presented case showed an atypical behavior of CLL with loss of CD19 and gain of features of plasma cells differentiation with a much more aggressive phenotype. Importantly, this clone was undistinguishable by routine flow cytometry at time of diagnosis but was detected by single-cell transcriptome profiling. This demonstrates the benefit of dissecting phenotypic heterogeneity by virtue of single-cell approaches. If translated to the clinic, such strategy can help characterizing the resistance potential of different clones at diagnosis that might otherwise not be recognized and in turn guide treatment decisions.

## Data Availability Statement

The datasets presented in this study can be found in online repositories. The names of the repository/repositories and accession number(s) can be found below: https://www.ncbi.nlm.nih.gov/geo/, GSE150930.

## Ethics Statement

The studies involving human participants were reviewed and approved by Joint ethic committee of Faculty Hospital Pilsen and Medical Faculty in Pilsen. The patients/participants provided their written informed consent to participate in this study. Written informed consent was obtained from the individual(s) for the publication of any potentially identifiable images or data included in this article.

## Author Contributions

Conceptualization: MH, PP, IB, and VC. Methodology: PP, HR, and VC. Formal analysis: PO, AD, and IB. Investigation: HR, KT, and VC. Resources: DL, JA, LH, and MH. Data curation: IB and PO. Writing—original draft preparation: MH, IB, PO, and VC. Visualization: IB, PO, and MH. Supervision: MH, IB, and VC. Funding acquisition: PP, JA, and LH. All authors contributed to the article and approved the submitted version.

## Funding

This research was supported by the National Sustainability Program I (NPU I) Nr. LO1503 provided by the Ministry of Education Youth and Sports of the Czech Republic, by the Charles University Research Fund (project number Q39). PO was funded by ELIXIR and the Czech the Bone Marrow Transplantation Foundation. IB was funded by an Imperial College Research Fellowship. VC was funded by Bloodwise (now Blood Cancer United Kingdom).

## Conflict of Interest

The authors declare that the research was conducted in the absence of any commercial or financial relationships that could be construed as a potential conflict of interest.
