# Supplementary material for: Evolution of Advanced Chronic Lymphoid Leukemia Unveiled by Single-Cell Transcriptomics: A Case Report
Source: Front Oncol. 2020 Oct 30;10:584607. doi: 10.3389/fonc.2020.584607 (PMC7664833; doi:10.3389/fonc.2020.584607)
Supplement: Supplementary file 1 [file DataSheet_1.pdf]

Supplementary information

Supplementary methods

Detailed methods used for sample collection, processing and analysis.

The file describes both wet lab as well as in silico methods.

File S1

R notebook containing the code used for data analysis and creation of the figures used in this publication.

The notebook contents code used or generation of most figures in this paper with exceptions of Figure 1, Figure 2A, Figure 3A and Figure S1E and tables with exception of Table S3.

Figure S1

Additional supporting figures

(a) clusters identified considering all cells profiled; (b) probabilistic cell types assigned to the clusters shown in (a); (c) summary of inferCNV results; while the upper heat map shows estimated CNAs in reference cells (normal B cells), the lower heat map indicates the same information for the captured, CLL cells; cells are shown on the rows, genomic position on the columns; (d) stacked bar charts indicating the relative cluster composition of the cells sampled either at diagnosis or relapse, after annotation to the most similar B cell stage of differentiation; (e) Survival curves stratified by high vs low expression of PARP14 (data and statistics from PRECOG).

Table S1

Statistics of the individual cells captured

For each cell, the % of reads mapping to the mitochondrial genome along with the number of unique transcripts and genes detected is indicated.

Table S2

Differentially expressed genes between the relapse-enriched and the diagnosis-enriched clusters

For each gene, the log2-fold-change, the fraction of cells showing expression in the two clusters and the adjusted p-value is shown

Table S3

Significantly enriched pathways in DEGs between the relapse-enriched and the diagnosis-enriched clusters (related to Fig. 3A)

For each significant set from Reactome, the ID along with a description, a FDR the list of DEGs annotated in that specific Reactome pathway are indicated

Table S4

Differentially expressed pathways between the relapse-enriched and the diagnosis-enriched clusters (related to Fig. 3B)

Table S5

Differentially expressed genes between REL1 and REL2

For each gene, the log2-fold-change, the fraction of cells showing expression in the two clusters and the adjusted p-value is shown
