# Supplementary material for: Evolution of Advanced Chronic Lymphoid Leukemia Unveiled by Single-Cell Transcriptomics: A Case Report
Source: Front Oncol. 2020 Oct 30;10:584607. doi: 10.3389/fonc.2020.584607 (PMC7664833; doi:10.3389/fonc.2020.584607)
Supplement: Supplementary file 2 [file DataSheet_2.pdf]

Materials and Methods

Sample acquisition and processing

Heparinized blood was collected, and peripheral blood mononuclear cells (PBMCs) were isolated by Ficoll density-gradient centrifugation (Ficol-Paque, GE Healthcare, 1000g/15min/dec and acc 0). In the diagnostic sample, the CLL cells were purified by negative selection using CLL isolation kit (Miltenyi, Germany) with anti CD2, CD3, CD4, CD14, CD15, CD16, CD34, CD56, CD61, CD235a (Glycophorin A) and FceRIa microbeads. The original representation of CLL was 90%. The sample was originally used for the testing of immunotherapy where infiltration with T cells could compromise the results, therefore CLL cells were purified before cryopreservation. The sample was included to this study to compare it to a very unique CD19 negative relapse. The relapsed PBMCs were stored without CLL purification. All cells were cryopreserved with 10% DMSO/FBS and stored in liquid nitrogen until the time of analysis. The cryopreserved PBMCs were thawed in a water bath at 37 °C and resuspended in warmed RPMI medium (Sigma). Cell concentration and viability was determined using a LUNA™ cell counter (Logos Biosystems) and their viability was quantified.

Flow cytometry

The isolated cells (in case of diagnosis the purified CLL, in case of relapse PBMCs) or peripheral blood were used for the evaluation of CLL immunophenotype. The cells were stained by the panel of the monoclonal antibodies: CD45-Krome Orange (clone J33), CD19-PC7 (clone J3-119), CD23-FITC (clone 9P25) , CD20-PE (clone B9H9) (all Beckman Coulter, France) or CD20-Pacific Blue (clone 2H7), CD5-PerCP-Cy5.5 (clone L17F12) (both Exbio, Czech Republic), CD200-APC (clone OX-104) (Biolegend, UK). The negative expression of CD19 was verified by different antibody (CD19-BB515, clone HIB 19, BD Bioscience). The antibodies were added to 100 µl of cell suspension or blood and incubated for 15 min in the dark at room temperature (RT)

followed by washing with 2 ml PBS (local source) for 5 min at 350g. In case of peripheral blood, red blood lysis with 1x BD FACS™ lysing solution (2ml, 10min; BD) was performed before washing step. The cell pellets were resuspended in 0.5 ml of PBS and immediately measured on a BD FACSCanto™ II (BD Bioscience) or NAVIOS (Beckman Coulter) cytometer. The analysis was performed using the FlowJo Software (v10.6.2; BD).

Cell hashing

Cell hashing was carried out prior to pooling and running on the 10x Chromium. Viability was found to be > 90% for both samples. One million cells from each sample were separately suspended in 100 µl cell staining buffer (BioLegend). The cells were blocked by incubating the samples at 4 °C for 10 min with 5 µl of Human TruStain FcX™ Fc blocking reagent (Biolegend), subsequently the samples were labelled by incubating the samples at 4 °C for 30 minutes with 1 µg of TotalSeq™-A0251 Hashtag 1 (cat. No. 394601; Biolegend; barcode; GTCAACTCTTTAGCG) or TotalSeq™-A0252 Hashtag 2 antibody (cat. No. 394603; Biolegend; barcode; TGATGGCCTATTGGG) for diagnosis and relapse, respectively. Cells were then washed with 1 mL of cell staining buffer (Biolegend) three times, resuspended in PBS at a concentration of 1000 cells/µl and filtered through 40 µm strainers to ensure single cell suspension. Cell concentration and viability were then verified using the LUNA™ cell counter (Logos Biosytems). The two samples were pooled at a 50:50 ratio and loaded onto the Chromium instrument as detailed by the manufacturer.

Single-cell RNA-sequencing library construction

Single cells were isolated and barcoded using the Chromium Single-Cell 3' Gene Expression Kit, version 3 chemistry (10x Genomics). The first step was to prepare the reverse transcription (RT) master mix as per manufacturer's instructions. Next, the Chromium Chip B was prepared and assembled in a chip holder, with 50% glycerol dispensed in any unused chip wells. The pre-prepared RT master mix was then added to the cell suspension before being loaded into row 1

without introducing any bubbles. Row 2 was loaded with gel beads, while row 3 was loaded with partitioning oil before a gasket was attached to the chip. The chip was then loaded into the Chromium controller (10x Genomics) to generate single-cell gel beads in emulsion (GEMs). The GEMs were aspirated from the recovery well and dispensed into a tube strip on ice. GEM-RT was carried out in a C1000 Touch Thermal cycler (Bio-Rad) as follows: 53 °C for 45 minutes, 85 °C for 5 minutes; held at 5 °C. Following RT, the GEMs were broken using a recovery agent and the single-strand cDNA was cleaned up with DynaBeads MyOne Silane Beads (Thermofisher) and subsequently washed twice with 80% ethanol before elution. cDNA was amplified using the 10x Amp mix and cDNA primers. Amplification was performed in a C1000 Touch Thermal cycler (Bio-Rad) as follows: 98 °C for 3 min; cycled 13x as follows: 98 °C for 15s, 63 °C for 20s, and 72 °C for 60s; 72 ^{o}C for 60s; held at 4 °C. Amplified cDNA product was cleaned up using SPRIselect Reagent Kit (0.6X SPRI; Beckman Coulter) before quantification on TapeStation (Agilent). The cDNA was subsequently fragmented to ~ 200 bp using the 10x Genomics fragmentation enzyme while run in a thermocycler at 32 °C for 5 min. End repair and A-tailing were then carried out in the C1000 Touch Thermal cycler (Bio-Rad) at 65 °C for 30 min. Double-sided size selection was then carried out using SPRIselect reagent. Adaptor ligation was carried out using adaptor oligos and ligation buffer provided by the Chromium Single-Cell 3' Library Kit (10x Genomics) and run in the thermocycler at 20 °C for 15 min before a 4 °C hold. This was followed by post-ligation clean-up with SPRIselect reagent. Finally, sample index PCR was carried out using a Chromium i7 sample index, amplification mix and primers provided by the Chromium library kit. The PCR was carried out in the C1000 Touch Thermal cycler (Bio-Rad) as follows: 98 °C 45s; cycled 16x: 98 °C for 20s, 54 °C for 30s, and 72 °C for 20s; 72 °C 60s; held at 4 °C. This was followed by one final clean-up using SPRIselect reagent. The barcode sequencing library was then quantified using TapeStation (Agilent) before sequencing.

Hashtag amplification and sequencing

At the cDNA amplification step, additional primers were added to increase yield of Hashtag oligonucleotide (HTO, cDNA derived from the TotalSeq™ Hashtag antibodies) products. 1 µl of HTO primer (5'GTGACTGGAGTTCAGACGTGTGC*T*C) was added and the cDNA amplification was carried out. Following cDNA amplification, 0.6X SPRI selection was performed to separate mRNA-derived and antibody-oligo-derived cDNAs, with the supernatant containing HTO-derived cDNAs (180 bp). The bead fraction containing the mRNA-derived cDNA (>300 bp) was washed with 80% ethanol and cDNA library preparation was carried out as described by manufacturer. The HTOs in the supernatant fraction were then purified using 2X SPRIselect purification before washing with 80% ethanol and subsequent elution in nuclease free water. HTO sequencing libraries were amplified using a separate PCR reaction. HTO reaction, 100 µl master mix contained: 45 µl purified Hashtag fraction, 50 µl 2x KAPA HiFi PCR Master Mix, 2.5 µl Truseq DNA D70x_s primer (containing i7 index) 10 µM, 2.5 µl SI PCR oligo. Cycling conditions were set as follows: 95 °C 3 min; cycled 12x: 95 °C 20 s, 64 °C 30s, 72 °C 20s; 72 °C 60s; held at 4 °C. Following amplification, the HTO products was purified using 1.6X SPRI purification. Both libraries (cDNA and HTO) were quantified using BioAnalyzer (Agilent), before being pooled in the following molar proportions: HTO libraries 10% and cDNA libraries 90%. The pooled libraries were then sequenced using Illumina NextSeq500.

sc-RNA-seq data analysis

Illumina generic adapters were removed from the fastq paired end reads during the base calling step using the bcl2fastq tool. CellRanger count was used to identify cell barcodes and quantify unique transcript counts according to GRCh38 annotation. After removing R2 reads shorter than 25 bps, CITE-seq-Count was used to quantify cell hashing data per lane using the cell barcodes reported by CellRanger. CellRanger was then re-run separately on the extracted reads for the two individual samples (diagnosis and relapse).

Hdf5 data for diagnosis and relapse were then load into the the statistical computing environment R using *Read10X_h5* function from Seurat package (1), merged by genes and turned into Seurat object by *CreateSeuratObject*. Percent of mitochondrial reads for each cell was determined by *PercentageFeatureSet* using ^MT- pattern. These genes were subsequently removed before further processing. Cells showing less than 1500 expressed transcripts, less than 500 expressed genes or more than 20% of reads mapping to mitochondrial genes were removed. Data were normalized by *SCTransform (2)* and principal components calculated using *RunPCA*. Clustering was peformed using the first 15 identified principal components, with default parameters. The number of components to include was evaluated using the elbow method. For further dimensionality reduction UMAP (3) was applied, using the *RunUMAP* function.

To annotate the identified clusters to known cell types signatures derived from (4) were used. The top 100 marker genes for each cell type were selected and used to calculate cell-type specific expression scores for each cell. Scores were computed using *AddModuleScore* from Seurat, and each cell was classified according to the signature with the highest score. Finally, the most common cell type assigned to the cells in each cluster was determined and used to classify the cluster.

After removing cells from clusters that were not containing B cells, the raw counts for the reaming cells were normalized, dimensionality reduced and clustered in the same way as described above for the full dataset, with exception that only first 12 principal components were considered.

B cell stage was assigned to each cell again using *AddModuleScore* and then selecting signature with highest score. Signatures were in this case derived from GenomicScape (5) and significance of differential proportion of stages calculated using *fisher.test*.

To identify CNAs, the function *infercnv* from the inferCNV package (6) was used with default settings except for *cutoff* set to 0.1 (as recommended for 10x data). As reference we used B cells from the 3kpbmc dataset provided by 10x Genomics (http://support.10xgenomics.com/single-cell-

gene-expression/datasets). B cells in this dataset were identified as described above. To obtain average CNAs for each cluster, the result table from *infercnv* (infercnv.preliminary.observations.txt) was loaded into R, and the predicted CNA for each gene averaged across all the cells in the cluster and plotted using ComplexHeatmap (7).

To identify differentially expressed genes between clusters we used *FindMarkers* with default settings and *wilcox.test*. Resulting genes were then exported for EnrichmentMap v3.2.1 (8) analysis or used for enrichment analysis. The enrichment analysis was performed using the clusterProfiler R package (9) and Reactome pathways as gene sets. For the EnrichmentMap analysis the enriched gene sets were determined using g:Profiler (10) with its version of ReactomePathways according to (11) and then visualized Cytoscape (12). To visualize pathways scores across cells given a gene set, the *AddModuleScore* was used. Similarly, single cells were scored for expression of a previously published signature for resistance to combined Rituximab, Fludarabine and Cyclophosphamide treatment (13). In line with the scoring used in the original publication, the sum of expression values was used as proxy for resistance.

Unless specified otherwise, all the analyses were performed in the statistical computing environment R v3 (14). Plots were generated using the package ggplot2 (15).

References

1. Stuart T, Butler A, Hoffman P, Hafemeister C, Papalexi E, Mauck WM, et al. Comprehensive Integration of Single-Cell Data. Cell. 2019 Jun 13;177(7):1888-1902.e21.

2. Hafemeister C, Satija R. Normalization and variance stabilization of single-cell RNA-seq data using regularized negative binomial regression. Genome Biology. 2019 Dec 23;20(1):296.

3. McInnes L, Healy J, Melville J. UMAP: Uniform Manifold Approximation and Projection for Dimension Reduction. 2018 Feb 9 [cited 2020 May 22]; Available from: https://arxiv.org/abs/1802.03426v2

4. Newman AM, Liu CL, Green MR, Gentles AJ, Feng W, Xu Y, et al. Robust enumeration of cell subsets from tissue expression profiles. Nat Methods. 2015 May;12(5):453–7.

5. Kassambara A, Rème T, Jourdan M, Fest T, Hose D, Tarte K, et al. GenomicScape: An Easy-to-Use Web Tool for Gene Expression Data Analysis. Application to Investigate the Molecular Events in the Differentiation of B Cells into Plasma Cells. PLOS Computational Biology. 2015 Jan 29;11(1):e1004077.

6. Patel AP, Tirosh I, Trombetta JJ, Shalek AK, Gillespie SM, Wakimoto H, et al. Single-cell RNA-seq highlights intratumoral heterogeneity in primary glioblastoma. Science. 2014 Jun 20;344(6190):1396–401.

7. Gu Z, Eils R, Schlesner M. Complex heatmaps reveal patterns and correlations in multidimensional genomic data. Bioinformatics. 2016 15;32(18):2847–9.

8. Merico D, Isserlin R, Stueker O, Emili A, Bader GD. Enrichment Map: A Network-Based Method for Gene-Set Enrichment Visualization and Interpretation. PLOS ONE. 2010 Nov 15;5(11):e13984.

9. Yu G, Wang L-G, Han Y, He Q-Y. clusterProfiler: an R package for comparing biological themes among gene clusters. OMICS. 2012 May;16(5):284–7.

10. Raudvere U, Kolberg L, Kuzmin I, Arak T, Adler P, Peterson H, et al. g:Profiler: a web server for functional enrichment analysis and conversions of gene lists (2019 update). Nucleic Acids Res. 2019 02;47(W1):W191–8.

11. Reimand J, Isserlin R, Voisin V, Kucera M, Tannus-Lopes C, Rostamianfar A, et al. Pathway enrichment analysis and visualization of omics data using g:Profiler, GSEA, Cytoscape and EnrichmentMap. Nat Protoc. 2019 Feb;14(2):482–517.

12. Shannon P. Cytoscape: A Software Environment for Integrated Models of Biomolecular Interaction Networks. Genome Research. 2003 Nov 1;13(11):2498–504.

13. Herling CD, Coombes KR, Benner A, Bloehdorn J, Barron LL, Abrams ZB, et al. Time-to-progression after front-line fludarabine, cyclophosphamide, and rituximab chemoimmunotherapy for chronic lymphocytic leukaemia: a retrospective, multicohort study. Lancet Oncol. 2019 Nov;20(11):1576–86.

14. R Core Team. R: A Language and Environment for Statistical Computing [Internet]. Vienna, Austria: R Foundation for Statistical Computing; 2020. Available from: https://www.R-project.org/

1. Stuart T, Butler A, Hoffman P, Hafemeister C, Papalexi E, Mauck WM, Hao Y, Stoeckius M, Smibert P, Satija R. Comprehensive Integration of Single-Cell Data. *Cell* (2019) **177**:1888-1902.e21. doi:10.1016/j.cell.2019.05.031

2. Hafemeister C, Satija R. Normalization and variance stabilization of single-cell RNA-seq data using regularized negative binomial regression. *Genome Biology* (2019) **20**:296. doi:10.1186/s13059-019-1874-1

3. McInnes L, Healy J, Melville J. UMAP: Uniform Manifold Approximation and Projection for Dimension Reduction. (2018) Available at: https://arxiv.org/abs/1802.03426v2 [Accessed May 22, 2020]

4. Newman AM, Liu CL, Green MR, Gentles AJ, Feng W, Xu Y, Hoang CD, Diehn M, Alizadeh AA. Robust enumeration of cell subsets from tissue expression profiles. *Nat Methods* (2015) **12**:453–457. doi:10.1038/nmeth.3337

5. Kassambara A, Rème T, Jourdan M, Fest T, Hose D, Tarte K, Klein B. GenomicScape: An Easy-to-Use Web Tool for Gene Expression Data Analysis. Application to Investigate the Molecular Events in the Differentiation of B Cells into Plasma Cells. *PLOS Computational Biology* (2015) **11**:e1004077. doi:10.1371/journal.pcbi.1004077

6. Patel AP, Tirosh I, Trombetta JJ, Shalek AK, Gillespie SM, Wakimoto H, Cahill DP, Nahed BV, Curry WT, Martuza RL, et al. Single-cell RNA-seq highlights intratumoral heterogeneity in primary glioblastoma. *Science* (2014) **344**:1396–1401. doi:10.1126/science.1254257

7. Gu Z, Eils R, Schlesner M. Complex heatmaps reveal patterns and correlations in multidimensional genomic data. *Bioinformatics* (2016) **32**:2847–2849. doi:10.1093/bioinformatics/btw313

8. Merico D, Isserlin R, Stueker O, Emili A, Bader GD. Enrichment Map: A Network-Based Method for Gene-Set Enrichment Visualization and Interpretation. *PLOS ONE* (2010) **5**:e13984. doi:10.1371/journal.pone.0013984

9. Yu G, Wang L-G, Han Y, He Q-Y. clusterProfiler: an R package for comparing biological themes among gene clusters. *OMICS* (2012) **16**:284–287. doi:10.1089/omi.2011.0118

10. Raudvere U, Kolberg L, Kuzmin I, Arak T, Adler P, Peterson H, Vilo J. g:Profiler: a web server for functional enrichment analysis and conversions of gene lists (2019 update). *Nucleic Acids Res* (2019) **47**:W191–W198. doi:10.1093/nar/gkz369

11. Reimand J, Isserlin R, Voisin V, Kucera M, Tannus-Lopes C, Rostamianfar A, Wadi L, Meyer M, Wong J, Xu C, et al. Pathway enrichment analysis and visualization of omics data using g:Profiler, GSEA, Cytoscape and EnrichmentMap. *Nat Protoc* (2019) **14**:482–517. doi:10.1038/s41596-018-0103-9

12. Shannon P. Cytoscape: A Software Environment for Integrated Models of Biomolecular Interaction Networks. *Genome Research* (2003) **13**:2498–2504. doi:10.1101/gr.1239303

13. Herling CD, Coombes KR, Benner A, Bloehdorn J, Barron LL, Abrams ZB, Majewski T, Bondaruk JE, Bahlo J, Fischer K, et al. Time-to-progression after front-line fludarabine, cyclophosphamide, and rituximab chemoimmunotherapy for chronic lymphocytic leukaemia: a retrospective, multicohort study. *Lancet Oncol* (2019) **20**:1576–1586. doi:10.1016/S1470-2045(19)30503-0

14. R Core Team. *R: A Language and Environment for Statistical Computing*. Vienna, Austria: R Foundation for Statistical Computing (2020). Available at: https://www.R-project.org/

15. Wickham H. *ggplot2: Elegant Graphics for Data Analysis*. Springer-Verlag New York (2016). Available at: https://ggplot2.tidyverse.org
